# Regulation of TRAIL expression by PRAME and EZH2 as potential therapeutic target against solid tumors

**DOI:** 10.1186/1753-6561-7-S2-P10

**Published:** 2013-04-04

**Authors:** Barbara P Mello, Daniel D de Carvalho, Antonio HJFM Campos, Fernando A Soares, Gustavo P Amarante-Mendes

**Affiliations:** 1 Department of Immunology, Sao Paulo University, São Paulo, Brazil; 2 Department of Pathologic Anatomy, AC Camargo Hospital, São Paulo, SP, Brazil

## Background

TRAIL, a member of the TNF ligand family, was shown to selectively kill cancer cells and, therefore, to participate in the cell-mediated immunity against tumors. However, TRAIL is down-regulated in a variety of cancers. Furthermore, PRAME (preferentially expressed antigen of melanoma) is frequently over expressed in a wide variety of malignant diseases. It was shown that PRAME, in a complex with a member of the polycomb group, EZH2, can function as a transcriptional repressor of retinoic acid receptor. Interestingly, TRAIL expression can be positively regulated by retinoic acid. Previous studies performed by us revealed that TRAIL is down-regulated and PRAME is up-regulated during development of chronic myeloid leukemia (CML) and that their normal levels are restored after complete cytogenetic remission (CCR). There was a significant, negative correlation between the expression of PRAME and TRAIL in CML patients. Over expression of BCR-ABL in the acute promyelocytic leukemia cell line HL-60 increased the levels of PRAME and decreased the levels of TRAIL. Knocking-down of either PRAME or EZH2 in K562 CML cell line resulted in TRAIL up-regulation.

## Materials and methods

We are continuing this study in solid tumors and sarcomas, through qRT-PCR and tissue microarray (TMA) immunohistochemistry, using samples from human cell lines and cancer patients.

## Results

Using the publicly available Oncomine Research platform, we found that PRAME was up- and TRAIL was down-regulated in several cancers. Literature data were validated by TMA immunohistochemistry, in tumor samples from patients with lung, prostate, breast and kidney cancers, melanoma and sarcoma. We are performing qRT-PCR assays to validate deregulated mRNA expression in several tumor cell lines and primary patient samples.

## Conclusions

These initial data, showing PRAME overexpressed in tumors, accompanied by a decreased expression of TRAIL, corroborate our hypothesis that the presence of a complex consisting of PRAME and EZH2 is responsible for the negative transcriptional regulation of TRAIL in cancer.

## Financial support

FAPESP and CNPq.

